# Unveiling the effects of metabolites on the material properties of natural rubber by the integration of metabolomics and material characteristics

**DOI:** 10.1038/s41598-025-91631-7

**Published:** 2025-04-15

**Authors:** Nobuyuki Hiraoka, Shunsuke Imai, Shintaro Shioyama, Fuminori Yoneyama, Akio Mase, Yuko Makita

**Affiliations:** 1 Fundamental Material Development Laboratory, Sumitomo Riko Company Ltd, Aichi, 485- 8550 Japan; 2 Graduate School of Engineering, Maebashi Institute of Technology, Maebashi, Gunma 371-0816 Japan

**Keywords:** *Hevea brasiliensis*, Non-rubber components, Seasonal changes, Latex, Metabolites, Metabolomics, Engineering

## Abstract

Natural rubber (NR) is an important material with excellent physical properties. Unlike synthetic rubber from petroleum, NR contains non-rubber components such as proteins, lipids, and metal ions. The non-rubber components are known to affect the properties of NR. In this study, latex samples of *Hevea brasiliensis* were collected for nine months and their metabolites were comprehensively analyzed by mass spectrometry. NR was made from the same latex samples used for the mass spectrometry, and their vulcanization, tensile and thermal-aging properties were assessed. By using this approach of integrating metabolite and property data, we aim to clarify the influence of metabolites on the physical properties of NR. These results suggest that the metabolite composition in the latex and the NR properties changed seasonally. Correlation analysis between the metabolites and the properties of NR indicated that different metabolites affected different properties. A regression model of NR properties using metabolites as the explanatory variables suggests that about five metabolites need to be considered when examining the relationship between properties and metabolites. This method, which combines comprehensive analysis and characterization of NR, contributes to studies aimed at elucidating how the superior properties of NR are brought about.

**Supplementary Information:**

The online version contains supplementary material available at 10.1038/s41598-025-91631-7.

## Introduction

Natural rubber (NR) is an important plant-based material produced from the emulsion called latex collected from *Hevea brasiliensis*^1^. Unlike synthetic rubber, which is manufactured through chemical synthesis, NR contains biological components such as plant-derived proteins, lipids, and metal ions. They are called non-rubber components and are known as contributing factors to NR’s excellent elasticity and durability against abrasion and cracking^2^. However, the composition of these non-rubber components varies depending on factors that are challenging to control, such as season, habitat and weather, and can reduce the stability of the rubber quality^3^.

NR is an indispensable raw material in industry as a result of its properties, being used in tires and anti-vibration products for transportation equipment^4^. Therefore, it is imperative to understand the correlation between the composition of non-rubber components and the physical properties of NR to control the latter even when the composition of non-rubber components fluctuates. Many analyses and studies have been carried out on the relationship between the non-rubber components and NR properties. Globally-distributed NR is standardized in each NR-producing country, and is commonly defined by the amount of dirt, ash, nitrogen, and volatile components present, and is reflected in the grade of NR. The non-rubber components are often analyzed simultaneously with an evaluation of the material properties, such as vulcanizing and viscoelasticity. The relationships between non-rubber components and NR properties have been investigated. For example, Zhao et al. showed that Mg^2+^, Mn^2+^, or Fe^2+^ decreased the Wallace plasticity value, and Ca^2+^, Zn^2+^, or Mg^2+^ decreased the plasticity retention index (PRI)^5^. For both Wallace plasticity and PRI, higher values indicate better quality rubber. Rojruthai et al.. showed that Fe^2+^ or Cu^2+^ causes delayed vulcanization, reduced tensile strength, and decreased viscosity during long-term storage^6^. In addition, the relationships between the properties of NR and protein, organic solvent extracts, and sugar content have been reported. Zhou et al.. demonstrated that proteins and phospholipids form a nanomatrix structure, and removing them reduces the tensile stress of natural rubber^7^. Nimpaiboon et al.. showed that monosaccharides reduce the Mooney viscosity of deproteinized natural rubber and synthetic isoprene rubber, but do not cause a decrease in the viscosity in natural rubber due to the Maillard reaction with proteins^8^. Thuong et al.. showed that acetone extraction improves the strength of natural rubber before crosslinking^9^. However, these previous reports have targeted a limited number of components contained in latex, or the overall measurements, such as protein content, and do not differentiate between individual components. To our knowledge, there are few examples of analyses linking non-rubber components with the properties of NR. Comprehensive analyses have been conducted on *Hevea brasiliensis* primarily in the field of breeding^10^ or on materials across multiple scales in the field of materiomics^11,12^. However, there are few examples showing the integration of these.

The metabolome refers to the complete collection of all metabolites found in a target organism and metabolites are small molecules produced through metabolic processes. Examples of metabolites in this study include sugar phosphates, amino acids, nucleic acids, organic acids, vitamins, dipeptides, fatty acids, and steroid derivatives. Here, we propose an approach that combines the metabolome of latex with NR’s physical properties. By using this approach, we aim to clarify the effects of metabolites on the physical properties of NR. It will then be possible to apply these results to technologies that will improve NR and stabilize its quality.

Firstly, we collected latex samples each month of the year and analyzed the metabolites they contained, focusing on seasonal changes. Next, we evaluated the vulcanizing, tensile, and heat-aging properties of NR made from these same latexes. Vulcanizing properties refer to the speed and degree of vulcanization, tensile properties to the elasticity and durability when stretched, and heat-aging properties to the degree of deterioration under high temperature. These properties are the basic evaluation targets for anti-vibration rubber. Then, to confirm correlation between metabolites and physical properties, we compared the changes in specific metabolites in the latexes with the changes in the properties of NR. In addition, we attempted to examine the complex relationships between metabolites and physical properties using a regression model that regresses the NR properties using analytical values of the metabolites. As a result, we detected approximately 400 metabolites in latex samples from May 2022 to January 2023. We also found metabolites that had strong correlation with some of the evaluated properties. We then built regression models using the metabolites that explained the NR property measurements, estimated the complexity of the relationship, and considered which metabolites may be strongly related to each measurement.

## Results and discussion

### Changes of metabolites in latex between rainy season and dry season

**Fig. 1:**
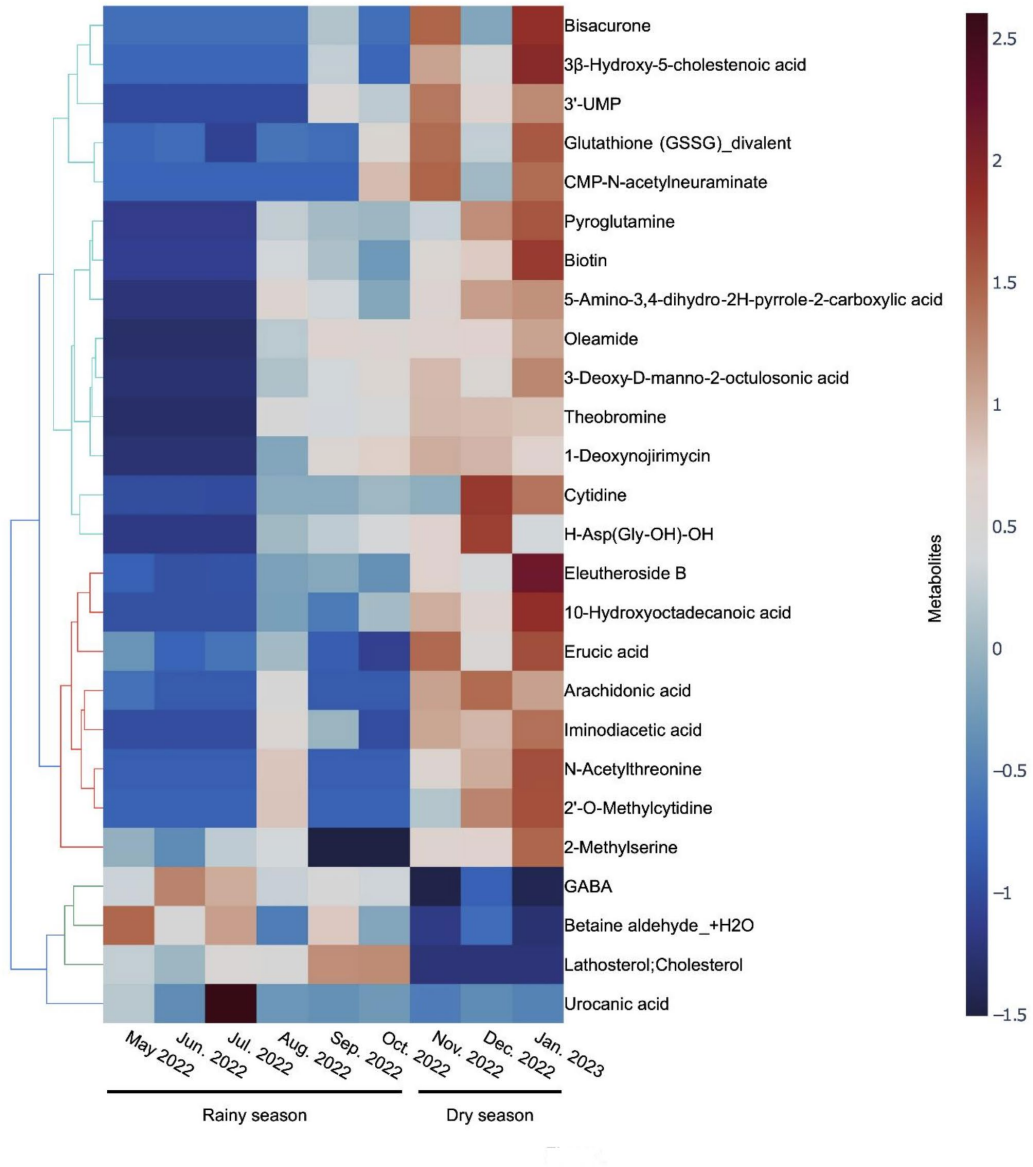
Metabolites exhibiting more than a two-fold difference between the mean values observed in the wet season and those observed in the dry season.

To identify metabolites affected by seasonal variations, liquid chromatography time-of-flight mass spectrometry (LC-TOFMS) and capillary electrophoresis time-of-flight mass spectrometry (CE-TOFMS) were used to determine the metabolite composition of the latex. The analysis was conducted on a total of nine samples, collected once a month from May 2022 to January 2023. Table 1 shows the number of identified peaks from the analysis. In three samples from May to July 2022, metabolites were identified in 375 peaks of MS. In six samples from August 2022 to January 2023, metabolites were identified in 389 peaks of MS. In the entire nine samples, a total of 431 peaks were annotated with metabolites but 25 of these peaks were annotated with more than two metabolites. Of the 431 peaks, 216 were detected in all nine samples. The concordance rate of identified metabolites between samples collected in different months was between 74% and 93% (Figure S1). Detected metabolites were classified based on chemical taxonomy information of the HMDB (Human Metabolome Database)^13^ (Figure S2). The metabolites were classified into 44 classes, excluding 100 metabolites for which class information was not available. Similarly, the metabolic pathways to which the metabolites belonged were confirmed, excluding 200 metabolites for which pathway information was not available. Forty-six pathways were listed, excluding 24 pathways unlikely in plants (e.g., cancer) (Figure S3). All latex samples were derived from the *Hevea brasiliensis* RRIM 600 clone in the same plantation; they were collected from 15 to 20 trees. Results suggest that the effect of individual differences (differences in tree age, trunk thickness) are small and other environmental factors (temperature, precipitation, soil moisture, sunshine, etc.) cause these metabolite differences. Since commercial NR is made by processing latex through acid coagulation, water washing, smoking, etc., it is highly likely that the metabolites contained in latex are different from those in commercial NR. This difference should be considered when applying our analysis method to commercial NR. Chachoengsao province in eastern Thailand has a savannah climate and is generally considered to have a warm rainy season from May to October, a cold dry season from November to February, and a hot rainy season from March to April. Plant metabolites are known to fluctuate with abiotic stresses such as temperature and water content, and seasonal variations have been reported for metabolites of other latex-producing plants such as guayule (*Parthenium argentatum*) and dandelion (*Taraxacum officinale*)^14–16^. A quantitive comparison of six samples from the wet season and three samples from the dry season revealed that four metabolites had a mean value more than two times lower than the mean value of the wet season and 22 metabolites had a mean value more than two times higher than the mean value of the wet season (Mann–Whitney *U* test, one-tailed, *n* = 6 and 3, each *p*-value is listed in Table S1). Figure 1 shows standardized values of these metabolites. In a comparison between the wet season and the dry season, drought stress in the dry season seems to be characteristic. First, we considered the relationship between drought stress and the four metabolites that decreased during the dry season. Accumulation of GABA and cholesterol have been reported to improve drought-stress tolerance^17–20^. It has also been reported that they are involved in stomatal blockage caused by drought stress during the dry season in *Hevea brasiliensis*^21^. Therefore, it is possible that the amount of GABA and cholesterol in latex decreased because of increased accumulation in drought-sensitive parts of the plant, such as leaves. Betaine aldehyde is a precursor of betaine, which is known to be enriched in response to salt and drought stress^22^. In our results, betaine increased about 1.4-fold in the dry season (*p*-value = 0.023), so it is difficult to argue that the variation of betaine aldehyde is caused by drought stress. Urocanic acid is known to be resistant to UV radiation mainly in animals, but its function in plants has not been reported, and it is unlikely that it has a specific effect in the dry season, because it is prominent only in June in the rainy season. Next, we considered the relationship between drought stress and the 22 metabolites that increased during the dry season. Theobromine is a precursor to caffeine. It is reported that caffeine synthase is possibly downregulated by drought stress in leaves of *Coffea canephora*. This regulation seems to relate with increased theobromine in *Hevea brasiliensis*^23^. 3β-hydroxy-5-cholestenoic acid is synthesized by the oxidation of cholesterol. Since cholesterol increases resistance to drought stress, the detected amount of 3β-hydroxy-5-cholestenoic acid is considered to be affected by drought stress. However, if cholesterol is decreased in latex due to a change in localization as described above, an investigation into the factors responsible for the increase in 3β-hydroxy-5-cholestenoic acid in latex is needed. Overall, about half of the metabolites were not detected in at least one sample. Therefore, not only do the seasons bring about large changes in the quantities of metabolites in latex but also whether they are present or absent.

**Table 1:** Number of peaks annotated with metabolites in the mass spectrometry analysis.

	Samples	Number of detected peaks
Rainy season	May 2022	340
	Jun. 2022	329
	Jul. 2022	335
	Aug. 2022	326
	Sep.2022	340
	Oct. 2022	321
Dry season	Nov. 2022	330
	Dec. 2022	348
	Jan. 2023	343
	Detected from at least one sample	431
	Detected throughout rainy season	225
	Detected throughout dry season	295
	Detected from all samples	216
	Detected in only rainy season	74
	Detected in only dry season	4

### Visualization of metabolite diversity using descriptors of molecular structure

**Fig. 2:**
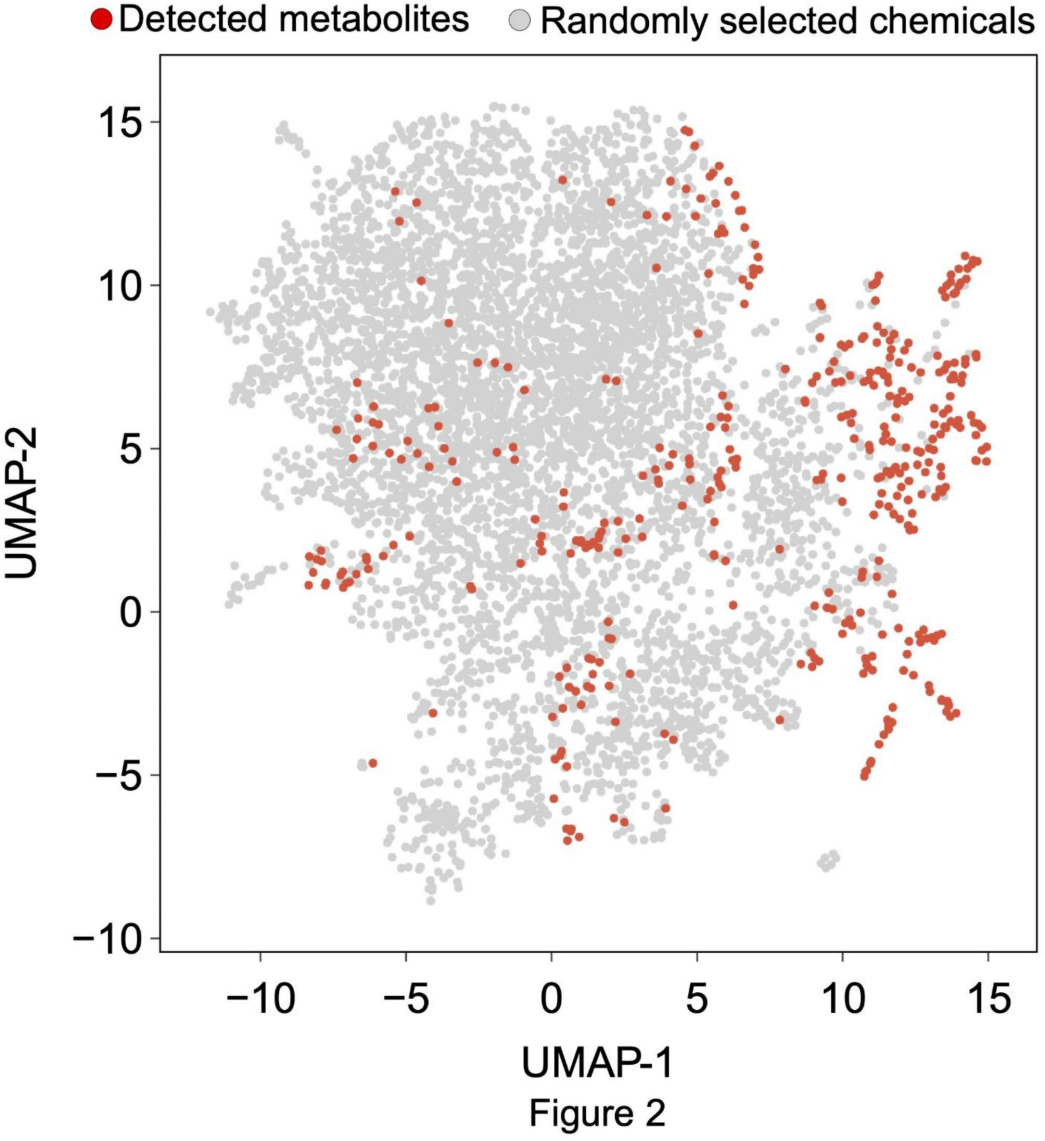
Structural diversity of metabolites. Plotted data was obtained by dimensionally compressing the Morgan fingerprint of each metabolite and randomly selected chemical compounds. UMAP-1 and UMAP-2 are two dimensions generated by UMAP (red circles: metabolites detected in latex; gray circles: 5,000 compounds randomly selected from PubChem).

To evaluate the effectiveness of the comprehensive analysis of metabolites, the diversity of metabolites detected in latex was evaluated using the Morgan fingerprint. The Morgan fingerprint is a collection of molecular features called descriptors that express the presence or absence of a specific substructure. Of the metabolites found in latex, 4096 descriptors were calculated for 406 metabolites. Figure 2 shows a scatter plot using Uniform Manifold Approximation and Projection (UMAP)^24,25^ to visualize the distance matrix of the 406 metabolites in red and 5000 compounds randomly selected from over 300,000 compounds with assigned chemical properties recorded in PubChem^26^ in gray. In addition, the random selection from PubChem was performed three times, one of which is shown in Fig. 2. In each of the three random compound selections, the coordinates on the UMAP plot were compared between 5000 compounds and the 406 detected in the latex. The results showed that there was a difference in the coordinates along either the UMAP-1 or UMAP-2 axis and no difference in the coordinates along the other axis in each of the three selections. However, the fact that the population of 406 metabolites was not ruled to be equal to that of the 5000 randomly selected metabolites (although only one of dimensions was compressed by UMAP) suggests that there is a certain amount of diversity of metabolites detected in the latex. Therefore, comprehensive analysis of latex containing metabolites with such diversity is effective when considering the effect of metabolites on NR properties.

### Properties of NR and correlation between the metabolites

To investigate the relationship between metabolites and NR properties, NR was prepared from the latex samples for a comprehensive analysis of metabolites. Characterization tests measured 30 items related to vulcanizing properties, 14 to tensile properties, and 36 to heat-aging properties. However, two items related to the heat-aging properties were excluded from subsequent analyses because the tests were completed before reaching the prescribed state and the values were left blank in all samples.

**Fig. 3:**
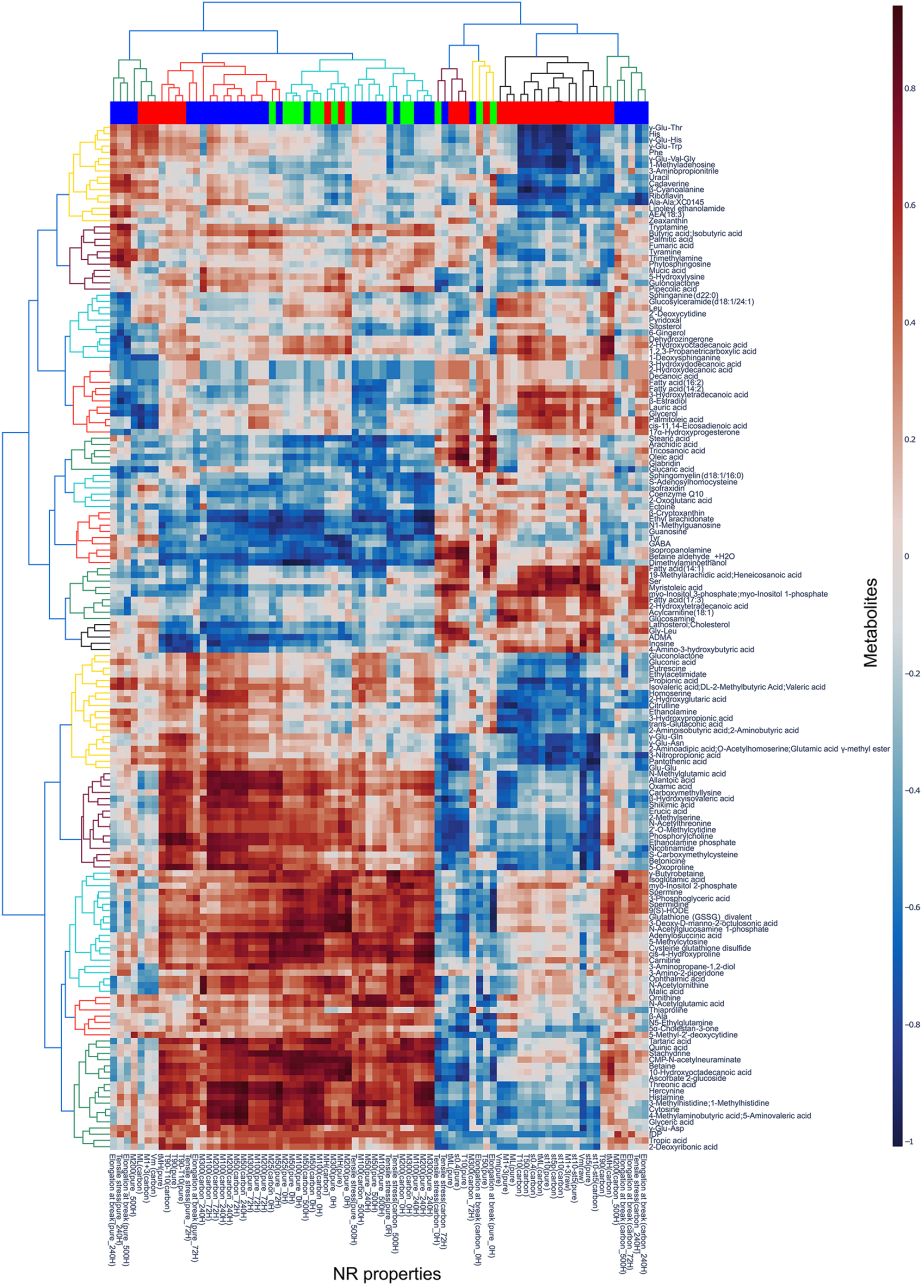
Hierarchical clustering of Spearman‘s rank correlation coefficient matrix between metabolites and NR properties. Colors of the horizontal color bar at the top of the heatmap indicate the test type (red: vulcanization; green: tensile; blue: heat-aging test) to which the NR properties belong.

To consider the effect of metabolites on property, correlation coefficients between metabolites and properties were calculated. The relative quantitative values of metabolites and NR properties were standardized. Spearman’s rank correlation coefficients were calculated between all metabolites and properties, as several items did not follow a normal distribution. Figure 3 shows the heatmap of the computed correlation coefficient matrix.

Vulcanizing, tensile and heat-aging properties represent three distinct types of evaluation. Therefore, we examined whether the correlations with metabolites differ in these three properties. The correlation coefficients related to vulcanizing properties were considered one group. Similarly, the correlation coefficients for tensile properties and those for heat-aging properties were each treated as separate groups, and the three groups were compared. In Fig. 3, the group to which each NR property belongs was indicated by the color bar at the top of the heat map (red: vulcanization, green: tensile, blue: heat-aging).

As a result, 165 metabolites had mean values for at least one group that differed significantly from other properties (Kruskal-Wallis test, one tailed, *n* = 30, 14, 34). For these 165 metabolites, the correlation matrix was visualized again by hierarchical clustering, and the metabolites were divided into two major groups. However, no obvious differences were observed between the two groups of metabolites in terms of molecular structure (data not shown).

### Complexity of the relationship between NR properties and metabolites

The complexity of the relationship between NR properties and metabolites is an important clue for elucidating these relationships. Here, complexity refers to the number of metabolites that need to be considered when explaining variations in a specific property. To quantify this complexity, the error of a linear regression model—where metabolites were used as explanatory variables for the properties—was analyzed. The evaluation method was based on the following idea: in the simplest case, the best regression model is constructed with a single metabolite, while in the most complex case, the best model requires all metabolites. For intermediate complexity, the optimal regression model would involve two or more metabolites but not all.

**Fig. 4:**
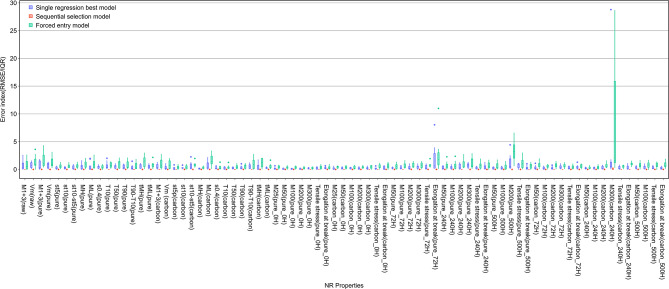
The relative predictive performance of various models for each measurement item. These nine values of RMSE/IQR for each item were calculated using leave-one-out cross-validation.

For each measurement item in the properties, three linear regression models were constructed with different combinations of metabolites as explanatory variables. The first model is the one with the smallest error among linear simple regression models using only one metabolite, hereafter referred to as the *simple regression best model*. The second is a linear multiple regression model that uses all the metabolites, referred to as the *forced entry model*. The third is a linear multiple regression model that uses multiple metabolites selected by the Sequential Forward Floating Selection (SFFS) method for each measurement as explanatory variables, the *sequential selection model*. The SFFS algorithm starts with 0 metabolites. At each step, the metabolite that minimizes the regression error of the model is selected from metabolites not included in the model. Subsequently, any metabolite whose elimination improves the model were removed. In this study, these additions and removals were repeated, and 10 metabolites were selected. Figure 4 shows the results of comparing the root mean square error / interquartile range (RMSE/IQR) of the three types of models mentioned above. Here, cross-validation was performed using the leave-one-out method on data from nine samples. RMSE/IQRs of the nine regression models were not averaged, and all values were visualized as a boxplot. As a result of testing the difference between the RMSE/IQRs of the simple regression best model and those of the sequential selection model, the RMSE/IQRs of the sequential selection model were significantly smaller for all items (Mann–Whitney *U* test, one-tailed, n = 9, each *p*-value is listed in Table S2). The RMSE for each item converged when five metabolites at most were selected for the sequential selection model (Figure S4). In considering the relationship between metabolites and the properties of NR, it is insufficient to consider only one metabolite, and excessive to consider more than about 10 metabolites. It may be most effective to consider five. Among the categories of the selected metabolites (Table S3), for all properties (vulcanization, tensile, and heat-aging), “carboxylic acids and derivatives,” “organonitrogen compounds,” and “fatty acyls” ranked in the top three and accounting for more than 50%. “keto acids and derivatives,” “organic phosphoric acids and derivatives,” and “glycerolipids” were only seen in the vulcanization properties. “cinnamic acids and derivatives” and “peptidomimetics” were only seen in the tensile properties. “sulfinic acids and derivatives,” “pyrroles,” “organic carbonic acids and derivatives,” and “isoflavonoids” were only seen in the heat aging properties. Regarding the pathways to which the selected metabolites belonged, most metabolites in the vulcanization and heat-aging properties were related to “lipid metabolism,” most metabolites in the tensile properties were related to “amino sugar metabolism.”

To investigate the possibility that the metabolites selected in the sequential selection model affect each property, up to five components for each measurement item were examined and the most overlapping metabolites were identified in each property.

**Fig. 5:**
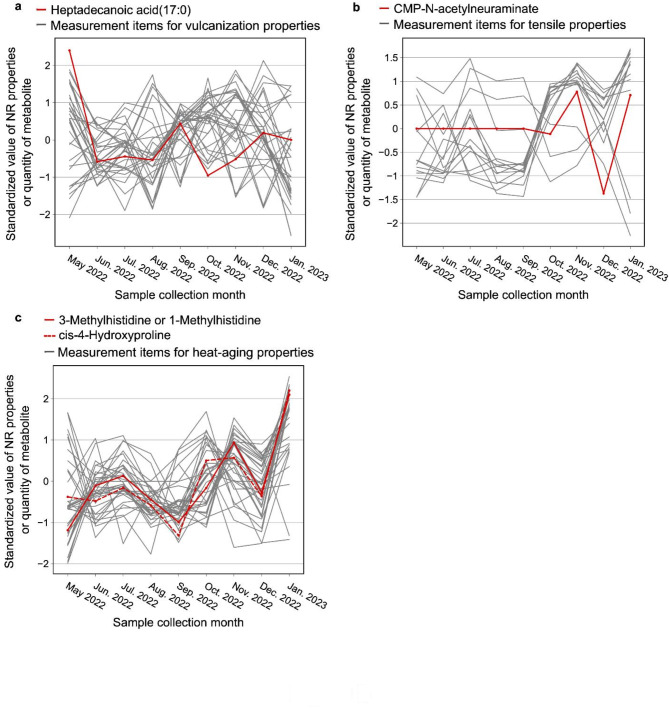
Standardized values of NR properties and most appeared metabolite in each kind of property.

Heptadecenoic acid (17:1) showed the highest overlap and appeared four times among the items related to vulcanizing properties. The test results of vulcanization properties and the measured values of heptadecenoic acid (17:1) are plotted together in Fig. 5(a). It is a fatty acid with 17 carbon atoms and one double bond. In sulfur vulcanization, which is a common method for vulcanizing NR, properties are generally adjusted by adding a vulcanization accelerator or a vulcanization retarder. Heptadecenoic acid (17:1) has a different structure from existing accelerators or retarders and is not considered to have the same function. On the other hand, it may act as a vulcanization accelerator aid, which is a component that activates a vulcanization accelerator. Zinc oxide (ZnO) is an essential vulcanization accelerator aid for sulfur vulcanization of NR and is included in this sample. Octadecanoic acid (18:0) is commonly used as an aid that reacts with ZnO and enhances its effect. This fatty acid has also been investigated for its influence on carbon number, and the longer the fatty chain length, the slower the vulcanization reaction^27^. Therefore, heptadecenoic acid (17:1) may act as a vulcanization accelerator, although further studies are needed because it is an unsaturated fatty acid. On the other hand, several effects of fatty acids on properties other than vulcanization properties have been reported, such as improvement of tensile properties of unvulcanized and vulcanized isoprene rubber^9,28,29^, and acceleration of oxidative degradation of NR depleted of endogenous proteins and fatty acids^30^.

In terms of tensile properties, CMP-N-acetylneuraminate showed the highest overlap and appeared seven times. The test results of tensile properties and the measured values of CMP-N-acetylneuraminate are plotted together in Fig. 5(b). This is a type of sugar nucleotide, and CMP is a common abbreviation for cytidine monophosphate. Tensile properties are generally adjusted by adding particles of carbon called carbon black, but CMP-N-acetylneuraminate is a very different substance from carbon black, so it is not expected to work the same way. It has also been reported that the tensile properties of NR are due to the nanocomposite of poly-isoprene with proteins and lipids^31^, but whether CMP-N-acetylneuraminate affects this nanocomposite has not been studied. Furthermore, it is known that plants do not produce CMP-N-acetylneuraminic acid or N-acetylneuraminic acid, so the CMP-N-acetylneuraminic acid detected here is suspected to be a contaminant. Therefore, the actual effect of CMP-N-acetylneuraminic acid on tensile properties should be carefully considered.

In terms of heat-aging properties, 1-methylhistidine or 3-methylhistidine and cis-4-hydroxyproline showed the highest overlap and appeared four times. The test results of heat-aging properties and the measured values of 1-methylhistidine or 3-methylhistidine, and cis-4-hydroxyproline are plotted together in Fig. 5(c). These are both amino acids; methylhistidine is a methylated histidine and hydroxyproline is a hydroxylated proline. Heat-aging properties are generally adjusted by the addition of antioxidants, but neither methylhistidine nor hydroxyproline has the structure necessary for the working mechanism of existing antioxidants. However, amino acids are known to act as natural antioxidants due to the presence of thiol or amino groups^32,33^. Therefore, it is possible that the amino groups of methylhistidine and hydroxyproline also have antioxidant effects. In addition, heat-aging was performed at 85 °C in this study, and the effects of metabolites may differ depending on the temperature. It is necessary to verify whether this difference will be a problem in future applications.

**Fig. 6:**
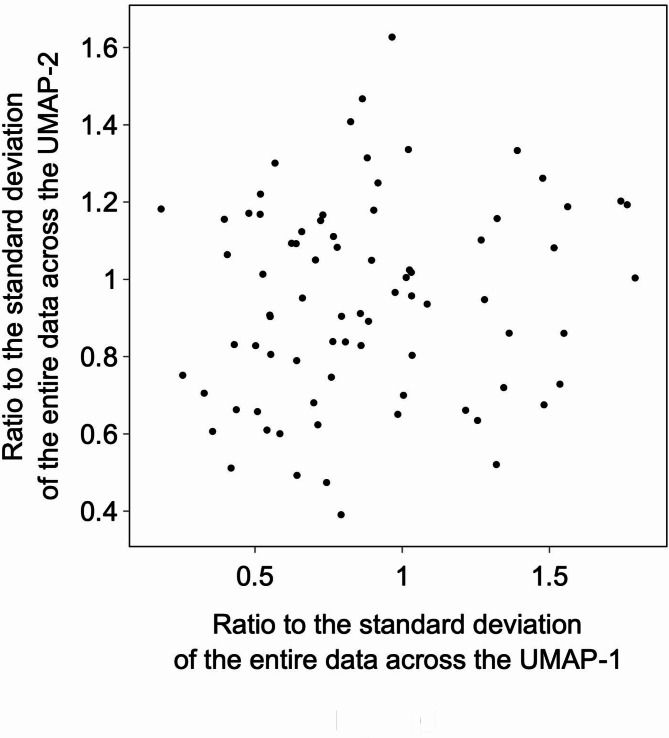
Structural diversity of metabolites selected by the sequential selection model for each physical property. Each axis indicates the diversity along each axis in Fig. 2.

We considered that if the metabolites selected in the sequential selection model are highly similar, experimental testing of all the compounds may not be necessary. Similarity was evaluated based on the molecular structure of each metabolite. The standard deviation (SD) of UMAP-1/UMAP-2 of all red markers shown in Fig. 2 was calculated (SD_all_). In the same way, the SD for metabolites selected by the sequential selection model was also calculated (SD_SS_). By comparing these two SDs, the diversity of metabolites was visualized. As shown in Fig. 6, the ratio between SDs (SD_SS_/SD_all_) varied depending on the measured item. This suggests that the diversity of the selected metabolites may be small, potentially reducing the number of metabolites needed to consider the relationship between metabolites and NR properties. However, this also raises the possibility that important metabolites with significant relationships might be overlooked if only a limited subset is considered.

## Conclusion

An integrated analysis of metabolomics and material properties for latex showed that the metabolite composition in latex and the properties of NR change with the season. Modeling and evaluation of the relationship between metabolites and the properties of NR suggested that most of the effects of metabolites on the properties could be explained by considering approximately five relevant metabolites.

By integrating the comprehensive analysis of the components of NR with its physical properties, the complex effects on physical properties of components other than those covered by the existing studies have been identified. Furthermore, the approach of modeling these effects will help to experimentally verify how NR’s excellent properties are brought about, and in the future, this will lead to the development of practical methods for improving and controlling NR properties.

## Materials and methods

### Latex sampling and storage

Latex was collected from 15 to 20 *Hevea brasiliensis* trees, aged between 10 and 20 years at the same plantation in Chachoengsao, Thailand. At the plantation, latex was collected every two days. Collection was suspended from February to April, but the samples in May were collected two weeks after collection resumed. The latex collected on same day was mixed. Then, to avoid coagulation, ammonia solution was added to the latex so that final concentration of ammonia was approximately 0.5wt%. After adding ammonia, the latex was divided into two portions, one for analysis of metabolites and the other for characterization of NR. The latex for analysis of metabolites was stored at 4 °C until analyzed. The latex for characterization of NR was added to an antiseptic agent (Bestcide-500, Nippon Soda Co., Ltd., Tokyo, Japan) and was stored at room temperature.

### Pretreatment for CE-TOFMS measurement

For analysis of metabolites using CE-TOFMS, metabolites were extracted in the following manner: 500 µL of methanol solution containing 50 µM of the internal standard was added to the latex sample, and the mixture was crushed with a bead shaker (Micro Smash, MS-100R, TOMY DIGITAL BIOLOGY CO., LTD., Tokyo, Japan) (3,500 rpm, 60 s x 20 times) under cooling. After crushing, 400 µL of Milli-Q water were added. The solution was stirred and then spun (2,300 × g, 4 °C, 5 min). The supernatant was transferred to an ultrafiltration tube (UltrafreeMC-PLHCC, HMT, centrifugal filter unit 5 kDa), centrifuged (9100×*g*, 4 °C, 120 min) and subjected to ultrafiltration. The filtrate was dried and dissolved in Milli-Q water again.

### Pretreatment for LC-TOFMS measurement

For analysis of metabolites using LC-TOFMS, metabolites were extracted in the following manner: 500 µL of 1% formic acid/acetonitrile containing 10 µM of the internal standard were added to the latex sample, and the mixture was crushed with the bead shaker (3,500 rpm, 60 s x 20 time) as above, under cooling. After crushing, 67 µL of Milli-Q water were added and the mixture was further crushed (3,500 rpm, 60 s x 1 time). The mixture was then centrifuged (2,300 × g, 4 °C, 5 min) and the supernatant was collected. The precipitate was added to 500 µL of 1% formic acid/acetonitrile and 167 µL of Milli-Q water and stirred. After centrifugation, the supernatant was collected and mixed with the previous supernatant. The combined solution was transferred to an ultrafiltration tube (Nanosep 3 K Omega, Pall) and centrifuged (9,100 × g, 4 °C, 120 min). Phospholipids were then removed using solid-phase extraction. The filtrate was dried and dissolved in 50% isopropanol aqueous solution (v/v).

### Chemical analysis and annotation of metabolites

Comprehensive analysis of the metabolites in a certain amount of latex (97 ± 1 mg) was conducted according to the *Dual Scan* package provided by Human Metabolome Technologies, Inc. (HMT). In this package CE-TOFMS and LC-TOFMS were used^34,35^. Briefly, CE-TOFMS analysis was conducted using an Agilent CE capillary electrophoresis system equipped with an Agilent 6210 time-of-flight mass spectrometer (Agilent Technologies, Inc., Santa Clara, CA, USA). LC-TOFMS analysis was conducted with an Agilent 1200 HPLC pump and an Agilent 6210 time-of-flight mass spectrometer (Agilent Technologies). The systems were controlled by Agilent G2201AA ChemStation software version B.03.01 for CE (Agilent Technologies) and MassHunter for LC (Agilent Technologies). The spectrometer was scanned from m/z 50 to 1,000 and peaks were extracted using MasterHands, an automatic integration software (Keio University, Tsuruoka, Yamagata, Japan) in order to obtain peak information including m/z, peak area, migration time (MT) for CE-TOFMS and retention time (RT) for LC-TOFMS analyses^36^. Signal peaks corresponding to isotopomers, adduct ions, and other product ions of known metabolites were excluded, and the remaining peaks were annotated according to HMT’s metabolite database based on their m/z values and MTs or RTs. Areas of the annotated peaks were then normalized to internal standards and the sample amount to obtain relative levels of each metabolite.

### Characterization of NR

The latex for characterization of NR was diluted with pure water to reach a certain concentration of total solids and was added to sodium dodecyl sulfate (SDS), and the mixture was stirred. SDS is a surfactant and used to prevent the latex from coagulating during drying through its emulsifying effect. NR samples were made by drying the mixture at 180 °C, and then kneading it with some of the following additives: sulfur, sulfenamide vulcanization accelerator, stearic acid, naphthenic oil, antioxidant and carbon black. Two different rubber samples were prepared by selecting from these additives according to two different recipes for making rubber compound. The measurement procedure followed a specific JIS (Japanese Industrial Standards), which is described below.

Vulcanizing properties (based on JIS K6300-1, K6300-2): Mooney viscosity was determined by a Mooney viscometer (AM-4, Toyo Seiki Seisaku-sho, Ltd, Tokyo, Japan) at 121 ℃, rotor type of L; one piece per sample was tested. Curing tests were conducted by a rotorless rheometer (RLR-4, Toyo Seiki Seisaku-sho, Ltd., TOKYO, JAPAN) at a temperature of 150 ℃, at a stress range of 200 kgf・cm, and an amplitude angle of ± 3°, one piece per sample was tested. Tensile properties (based on JIS K6251): Tensile tests were conducted by an automatic rubber tensile tester (Strograph AE Elastomer, Toyo Seiki Seisaku-sho, Ltd, Tokyo, Japan) at a pull speed of 500 mm/min, and dumbbell shape No. 5; three pieces per sample were tested and median was taken. Heat-aging properties (based on JIS K6257, K6251): The samples were aged in air at 85℃ for 72, 240–500 h. After aging, a tensile test was conducted using the automatic rubber tensile tester, or a tensile and compression testing machine (Technograph TGI-1KN, MinebeaMitsumi Inc., Tokyo, Japan) depending on the samples’ condition; three pieces per sample were tested and median was taken. The measurement items in each test are shown in Table S3.

### Visualization of the structural diversity of metabolites

The structural diversity of metabolites detected from the latexes was visualized by compressing the Morgan fingerprint of each metabolite into two dimensions by the UMAP method. The Morgan fingerprint was determined using MOL format data computed from canonical SMILES included in PubChem, an open chemistry database at the National Institutes of Health (NIH). Note that metabolites without compound IDs in PubChem are omitted from the visualization due to the lack of normalized SMILES. MOL format data were computed from the canonical SMILES by the function *GetMorganFingerprintAsBitVect* within the module *Allchem* in the Python library *rdkit*. Morgan fingerprints were computed from MOL format data by the function *AddMoleculeColumnToFrame* within the module *PandasTools* in the same library. Canonical SMILES were obtained from the PubChem based on the Compound ID. Arguments for the UMAP method processed by the class *UMAP* within the Python library *umap-learn* are as follows; *n_components* was set to 2, *n_neighbors* to 3, *min_dist* to 0.5, *metric* to manhattan, and *random_state* to 42.

## Electronic supplementary material

Below is the link to the electronic supplementary material.

Supplementary Material 1

## Data Availability

The datasets of mass spectrometry generated and/or analyzed during the current study are available in Metabolomic Workbench[https://www.metabolomicsworkbench.org/], Project DOI: 10.21228/M8RN7X. The datasets of the characterization of the NR generated and/or analyzed during the current study are available from the authors on reasonable request.
