# Supplementary material for: Unveiling the effects of metabolites on the material properties of natural rubber by the integration of metabolomics and material characteristics
Source: Sci Rep. 2025 Apr 15;15:11341. doi: 10.1038/s41598-025-91631-7 (PMC12000568; doi:10.1038/s41598-025-91631-7)
Supplement: Supplementary file 1 — Supplementary Material 1 [file 41598_2025_91631_MOESM1_ESM.pdf]

Supplementary information for

# Unveiling the effects of metabolites on the material properties of natural rubber by the integration of metabolomics and material characteristics

**Nobuyuki Hiraoka[1,2], Shunsuke Imai[1], Shintaro Shioyama[1], Fuminori Yoneyama[1], Akio Mase[1] & Yuko Makita[2*]**

**[1]Fundamental Material Development Laboratory, Sumitomo Riko Company Ltd, Aichi, 485-8550, Japan**
**[2]Graduate School of Engineering, Maebashi Institute of Technology, Maebashi, Gunma, 371-0816, Japan**

Email address: makita@maebashi-it.ac.jp

The supplementary information contains supplementary tables and figures:

Supplementary Tables 1-4 (p. 3-25)

**Supplementary Table 1:** *p*-values for components with amounts that differed by more than two-fold between rainy season and dry season.

**Supplementary Table 2:** *p*-values of Mann-Whitney *U* test for RMSE/IQRs of the sequential selection model and the simple regression best model.

**Supplementary Table 3:** Classes of selected metabolites in sequential selection model. For items with more than five classes, metabolites that could not be distinguished by mass spectrometry were selected together and then classified separately.

**Supplementary Table 4:** Items measured in characterization of NR and the measurement methods used. The measurement method basically follows the JIS (Japanese Industrial Standards) below. Any changes from the JIS are noted.

Supplementary Figures 1-4 (p. 26-29)

**Supplementary Fig. 1:** Percentage of common peaks among the monthly results of metabolite analysis.

**Supplementary Fig. 2:** Classes of detected metabolites based on chemical taxonomy information from Human Metabolome Database.

**Supplementary Fig. 3:** Metabolic pathways the detected metabolites belong to, according to metabolic pathway information from the Human Metabolome Database.

**Supplementary Fig. 4:** Transition of error values during the variable selection process in the sequential selection model, the legend shows only the top 10 average error values when there are 1 to 10 variables.

**Supplementary Table 1:** *p*-values for components with amounts that differed by more than two-fold between rainy season and dry season.

| Compound name | *p*-value | $Log_2$(Rainy season / Dry season) |
|---|---|---|
| 10-Hydroxyoctadecanoic acid | 0.026 | -2.5865 |
| 1-Deoxynojirimycin | 0.049 | -1.38225 |
| 2'-O-Methylcytidine | 0.034 | -2.75652 |
| 3-Deoxy-D-manno-2-octulosonic acid | 0.049 | -1.39915 |
| 3'-UMP | 0.022 | -2.18066 |
| 3β-Hydroxy-5-cholestenoic acid | 0.016 | -3.51146 |
| 5-Amino-3,4-dihydro-2H-pyrrole-2-carboxylic acid | 0.049 | -1.53801 |
| Biotin | 0.026 | -1.85176 |
| Bisacurone | 0.034 | -3.66355 |
| CMP-N-acetylneuraminate | 0.034 | -2.67202 |
| H-Asp(Gly-OH)-OH | 0.049 | -1.58813 |
| Iminodiacetic acid | 0.022 | -2.3401 |
| N-Acetylthreonine | 0.034 | -2.80707 |
| Oleamide | 0.026 | -1.23566 |
| Pyroglutamine | 0.026 | -1.79374 |
| Theobromine | 0.026 | -1.33251 |
| Arachidonic acid | 0.022 | -2.99541 |
| Cytidine | 0.048 | -2.08092 |
| Erucic acid | 0.024 | -1.32095 |
| 2-Methylserine | 0.028 | -1.24697 |
| Eleutheroside B | 0.024 | -1.66562 |
| Glutathione (GSSG)_divalent | 0.048 | -1.92316 |
| Lathosterol;Cholesterol | 0.026 | 36.46075 |
| Urocanic acid | 0.024 | 1.689232 |
| Betaine aldehyde_+H2O | 0.024 | 2.077382 |
| GABA | 0.024 | 2.193538 |

**Supplementary Table 2:** *p*-values of Mann-Whitney *U* test for RMSE/IQRs of the sequential selection model and the simple regression best model.

| Properties | *p*-value |
|---|---|
| M1+3(raw) | 0.000206147 |
| Vm(raw) | 0.000206147 |
| M1+3(pure) | 0.000206147 |
| Vm(pure) | 0.000206147 |
| st5(pure) | 0.000206147 |
| st10(pure) | 0.000206147 |
| st10-st5(pure) | 0.000206147 |
| MH(pure) | 0.000206147 |
| ML(pure) | 0.000286817 |
| s0.4(pure) | 0.000206147 |
| T10(pure) | 0.000206147 |
| T50(pure) | 0.000206147 |
| T90(pure) | 0.000206147 |
| T90-T10(pure) | 0.000396134 |
| tMH(pure) | 0.000206147 |
| tML(pure) | 0.000206147 |
| M1+3(carbon) | 0.000206147 |
| Vm (carbon) | 0.000206147 |
| st5p(carbon) | 0.000206147 |
| st10(carbon) | 0.000206147 |
| st10-st5(carbon) | 0.000206147 |
| MH(carbon) | 0.000206147 |
| ML(carbon) | 0.000206147 |
| s0.4(carbon) | 0.000206147 |
| T10(carbon) | 0.000206147 |
| T50(carbon) | 0.000206147 |
| T90(carbon) | 0.000206147 |
| T90-T10(carbon) | 0.000206147 |
| tMH(carbon) | 0.00020473 |
| tML(carbon) | 0.00020332 |
| M25(pure_0H) | 0.000206147 |
| M50(pure_0H) | 0.000206147 |

| **Properties** | ***p*-value** |
|---|---|
| M100(pure_0H) | 0.000206147 |
| M200(pure_0H) | 0.00020473 |
| M300(pure_0H) | 0.000206147 |
| Tensile stress(pure_0H) | 0.000206147 |
| Elongation at break(pure_0H) | 0.000206147 |
| M25(carbon_0H) | 0.000206147 |
| M50(carbon_0H) | 0.000206147 |
| M100(carbon_0H) | 0.000206147 |
| M200(carbon_0H) | 0.000206147 |
| M300(carbon_0H) | 0.000206147 |
| Tensile stress(carbon_0H) | 0.000206147 |
| Elongation at break(carbon_0H) | 0.000206147 |
| M50(pure_72H) | 0.000206147 |
| M100(pure_72H) | 0.000206147 |
| M200(pure_72H) | 0.000206147 |
| M300(pure_72H) | 0.000206147 |
| Tensile stress(pure_72H) | 0.000206147 |
| Elongation at break(pure_72H) | 0.000206147 |
| M50(pure_240H) | 0.000206147 |
| M100(pure_240H) | 0.000206147 |
| M200(pure_240H) | 0.000206147 |
| M300(pure_240H) | 0.000206147 |
| Tensile stress(pure_240H) | 0.000206147 |
| Elongation at break(pure_240H) | 0.000206147 |
| M50(pure_500H) | 0.000206147 |
| M100(pure_500H) | 0.000206147 |
| M200(pure_500H) | 0.000206147 |
| Tensile stress(pure_500H) | 0.000206147 |
| Elongation at break(pure_500H) | 0.000206147 |
| M50(carbon_72H) | 0.000206147 |
| M100(carbon_72H) | 0.000206147 |
| M200(carbon_72H) | 0.000206147 |
| M300(carbon_72H) | 0.000206147 |
| Tensile stress(carbon_72H) | 0.000206147 |
| Elongation at break(carbon_72H) | 0.000206147 |

| Properties | *p*-value |
|---|---|
| M50(carbon_240H) | 0.000206147 |
| M100(carbon_240H) | 0.000206147 |
| M200(carbon_240H) | 0.000206147 |
| M300(carbon_240H) | 0.000396134 |
| Tensile stress(carbon_240H) | 0.000206147 |
| Elongation at break(carbon_240H) | 0.00020473 |
| M50(carbon_500H) | 0.000206147 |
| M100(carbon_500H) | 0.000206147 |
| Tensile stress(carbon_500H) | 0.000206147 |
| Elongation at break(carbon_500H) | 0.000206147 |

**Supplementary Table 3:** Classes of selected metabolites in sequential selection model. For items with more than five classes, metabolites that could not be distinguished by mass spectrometry were selected together and then classified separately.

| measured items | Classes of each metabolite selected in sequential selection model |
|---|---|
| M1+3(raw) | Carboxylic acids and derivatives<br>Azoles<br>Indoles and derivatives<br>Organonitrogen compounds<br>Sphingolipids |
| Vm(raw) | Carboxylic acids and derivatives<br>Carboxylic acids and derivatives<br>Steroids and steroid derivatives<br>Carboxylic acids and derivatives<br>Steroids and steroid derivatives |
| M1+3(pure) | Carboxylic acids and derivatives<br>Prenol lipids<br>Organonitrogen compounds<br>Carboxylic acids and derivatives<br>Benzene and substituted derivatives |

| measured items | Classes of each metabolite selected in sequential selection model |
|---|---|
| Vm(pure) | Carboxylic acids and derivatives<br>Carboxylic acids and derivatives<br>Benzene and substituted derivatives<br>Organonitrogen compounds<br>Organooxygen compounds<br>Carboxylic acids and derivatives |
| st5(pure) | Fatty Acyls<br>Carboxylic acids and derivatives<br>Carboxylic acids and derivatives<br>Flavonoids<br>Carboxylic acids and derivatives |
| st10(pure) | Fatty Acyls<br>Carboxylic acids and derivatives<br>Fatty Acyls<br>Imidazopyrimidines<br>Carboxylic acids and derivatives |
| st10-st5(pure) | Piperidines<br>Pyrimidine nucleosides<br>Azoles<br>Organonitrogen compounds<br>Carboxylic acids and derivatives<br>Imidazopyrimidines |
| MH(pure) | Pyrimidine nucleosides<br>Fatty Acyls<br>Organonitrogen compounds<br>Carboxylic acids and derivatives<br>Keto acids and derivatives |
| ML(pure) | Ribonucleoside 3'-phosphates<br>Hydroxy acids and derivatives<br>Carboxylic acids and derivatives<br>Carboxylic acids and derivatives<br>Fatty Acyls |

| measured items | Classes of each metabolite selected in sequential selection model |
|---|---|
| s0.4(pure) | Piperidines<br>Pyrimidine nucleosides<br>Fatty Acyls<br>Fatty Acyls<br>Carboxylic acids and derivatives<br>Carboxylic acids and derivatives |
| T10(pure) | Steroids and steroid derivatives<br>Organonitrogen compounds<br>Fatty Acyls<br>Organooxygen compounds<br>Phenols |
| T50(pure) | Carboxylic acids and derivatives<br>Steroids and steroid derivatives<br>Carboxylic acids and derivatives<br>Carboxylic acids and derivatives<br>Lactones<br>Steroids and steroid derivatives |
| T90(pure) | Organonitrogen compounds<br>Carboxylic acids and derivatives<br>Purine nucleotides<br>Carboxylic acids and derivatives<br>Prenol lipids |
| T90-T10(pure) | Organonitrogen compounds<br>Pyrimidine nucleosides<br>Prenol lipids<br>Pyridines and derivatives<br>Pyrimidine nucleotides |
| tMH(pure) | Carboxylic acids and derivatives<br>Hydroxy acids and derivatives<br>Carboxylic acids and derivatives<br>Organonitrogen compounds<br>Carboxylic acids and derivatives |

| measured items | Classes of each metabolite selected in sequential selection model |
|---|---|
| tML(pure) | Fatty Acyls<br>Organooxygen compounds<br>Carboxylic acids and derivatives<br>Benzene and substituted derivatives<br>Steroids and steroid derivatives |
| M1+3(carbon) | Steroids and steroid derivatives<br>Carboxylic acids and derivatives<br>Organonitrogen compounds<br>Carboxylic acids and derivatives<br>Organonitrogen compounds |
| Vm (carbon) | Pyridines and derivatives<br>Organic phosphoric acids and derivatives<br>Organonitrogen compounds<br>Fatty Acyls<br>Carboxylic acids and derivatives |
| st5p(carbon) | Carboxylic acids and derivatives<br>Steroids and steroid derivatives<br>Carboxylic acids and derivatives<br>Organooxygen compounds<br>Fatty Acyls |
| st10(carbon) | Glycerophospholipids<br>Carboxylic acids and derivatives<br>Organooxygen compounds<br>Carboxylic acids and derivatives<br>Organonitrogen compounds |
| st10-st5(carbon) | Organonitrogen compounds<br>Quinolines and derivatives<br>Carboxylic acids and derivatives<br>Organonitrogen compounds<br>Carboxylic acids and derivatives |

| measured items | Classes of each metabolite selected in sequential selection model |
|---|---|
| MH(carbon) | Carboxylic acids and derivatives<br>Purine nucleotides<br>Glycerolipids<br>Organooxygen compounds<br>Fatty Acyls |
| ML(carbon) | Azoles<br>Carboxylic acids and derivatives<br>Flavonoids<br>Phenols<br>Carboxylic acids and derivatives |
| s0.4(carbon) | Fatty Acyls<br>Carboxylic acids and derivatives<br>Steroids and steroid derivatives<br>Sphingolipids<br>Carboxylic acids and derivatives |
| T10(carbon) | Carboxylic acids and derivatives<br>Fatty Acyls<br>Carboxylic acids and derivatives<br>Carboxylic acids and derivatives<br>Organonitrogen compounds |
| T50(carbon) | Carboxylic acids and derivatives<br>Fatty Acyls<br>Carboxylic acids and derivatives<br>Carboxylic acids and derivatives<br>Imidazopyrimidines |
| T90(carbon) | Pyrimidine nucleosides<br>Carboxylic acids and derivatives<br>Pyridines and derivatives<br>Steroids and steroid derivatives<br>Fatty Acyls |

| measured items | Classes of each metabolite selected in sequential selection model |
|---|---|
| T90-T10(carbon) | Fatty Acyls |
| | Fatty Acyls |
| | Organonitrogen compounds |
| | Keto acids and derivatives |
| | Carboxylic acids and derivatives |
| tMH(carbon) | Glycerophospholipids |
| | Organonitrogen compounds |
| | Flavonoids |
| | Prenol lipids |
| | Steroids and steroid derivatives |
| | Benzene and substituted derivatives |
| tML(carbon) | Ribonucleoside 3'-phosphates |
| | Flavonoids |
| | Purine nucleosides |
| | Carboxylic acids and derivatives |
| | Organonitrogen compounds |
| M25(pure_0H) | Fatty Acyls |
| | Fatty Acyls |
| | Imidazopyrimidines |
| | Carboxylic acids and derivatives |
| | Organonitrogen compounds |
| M50(pure_0H) | Pyrimidine nucleotides |
| | Fatty Acyls |
| | Carboxylic acids and derivatives |
| | Azoles |
| | Fatty Acyls |
| M100(pure_0H) | Carboxylic acids and derivatives |
| | Organonitrogen compounds |
| | Carboxylic acids and derivatives |
| | Carboxylic acids and derivatives |
| | Carboxylic acids and derivatives |

| measured items | Classes of each metabolite selected in sequential selection model |
|---|---|
| M200(pure_0H) | Carboxylic acids and derivatives<br>Organonitrogen compounds<br>Carboxylic acids and derivatives<br>Carboxylic acids and derivatives<br>Carboxylic acids and derivatives |
| M300(pure_0H) | Fatty Acyls<br>Carboximidic acids and derivatives<br>Carboxylic acids and derivatives<br>Fatty Acyls<br>Carboxylic acids and derivatives<br>Organooxygen compounds |
| Tensile stress(pure_0H) | Indoles and derivatives<br>Imidazopyrimidines<br>Flavonoids<br>Imidazopyrimidines<br>Hydroxy acids and derivatives |
| Elongation at break(pure_0H) | Carboxylic acids and derivatives<br>Carboxylic acids and derivatives<br>Carboxylic acids and derivatives<br>Organooxygen compounds<br>Phenols |
| M25(carbon_0H) | Fatty Acyls<br>Organonitrogen compounds<br>Cinnamic acids and derivatives<br>Steroids and steroid derivatives<br>Carboxylic acids and derivatives |
| M50(carbon_0H) | Organonitrogen compounds<br>Pteridines and derivatives<br>Carboxylic acids and derivatives<br>Prenol lipids<br>Purine nucleotides<br>Carboxylic acids and derivatives |

| measured items | Classes of each metabolite selected in sequential selection model |
|---|---|
| M100(carbon_0H) | Carboxylic acids and derivatives<br>Peptidomimetics<br>Carboxylic acids and derivatives<br>Prenol lipids<br>Organooxygen compounds |
| M200(carbon_0H) | Carboxylic acids and derivatives<br>Carboxylic acids and derivatives<br>Carboxylic acids and derivatives<br>Pyrimidine nucleosides<br>Benzene and substituted derivatives |
| M300(carbon_0H) | Carboxylic acids and derivatives<br>Carboxylic acids and derivatives<br>Carboxylic acids and derivatives<br>Carboxylic acids and derivatives<br>Purine nucleosides |
| Tensile stress(carbon_0H) | Carboxylic acids and derivatives<br>Prenol lipids<br>Organooxygen compounds<br>Carboxylic acids and derivatives<br>Carboxylic acids and derivatives |
| Elongation at break(carbon_0H) | Organonitrogen compounds<br>Carboxylic acids and derivatives<br>Prenol lipids<br>Purine nucleosides<br>Organonitrogen compounds |
| M50(pure_72H) | Phenols<br>Glycerophospholipids<br>Organonitrogen compounds<br>Pyrimidine nucleotides<br>Imidazopyrimidines |

| measured items | Classes of each metabolite selected in sequential selection model |
|---|---|
| M100(pure_72H) | Carboxylic acids and derivatives<br>Isoflavonoids<br>Carboxylic acids and derivatives<br>Fatty Acyls<br>Fatty Acyls |
| M200(pure_72H) | Sphingolipids<br>Organooxygen compounds<br>Carboxylic acids and derivatives<br>Organooxygen compounds<br>Carboxylic acids and derivatives<br>Fatty Acyls |
| M300(pure_72H) | Glycerophospholipids<br>Sphingolipids<br>Carboxylic acids and derivatives<br>Sphingolipids<br>Organooxygen compounds |
| Tensile stress(pure_72H) | Carboxylic acids and derivatives<br>Ribonucleoside 3'-phosphates<br>Carboxylic acids and derivatives<br>Carboxylic acids and derivatives<br>Carboxylic acids and derivatives |
| Elongation at break(pure_72H) | Fatty Acyls<br>Carboxylic acids and derivatives<br>Carboxylic acids and derivatives<br>Lactones<br>Fatty Acyls |
| M50(pure_240H) | Fatty Acyls<br>Sulfinic acids and derivatives<br>Fatty Acyls<br>Carboximidic acids and derivatives<br>Organooxygen compounds |

| **measured items** | **Classes of each metabolite selected in sequential selection model** |
|---|---|
| M100(pure_240H) | Carboxylic acids and derivatives<br>Carboximidic acids and derivatives<br>Steroids and steroid derivatives<br>Carboxylic acids and derivatives<br>Organonitrogen compounds |
| M200(pure_240H) | Carboxylic acids and derivatives<br>Carboxylic acids and derivatives<br>Fatty Acyls<br>Pyrimidine nucleosides<br>Carboxylic acids and derivatives |
| M300(pure_240H) | Carboxylic acids and derivatives<br>Fatty Acyls<br>Pyrimidine nucleosides<br>Carboxylic acids and derivatives<br>Pyrimidine nucleotides |
| Tensile stress(pure_240H) | Pyrroles<br>Prenol lipids<br>Fatty Acyls<br>Fatty Acyls<br>Carboxylic acids and derivatives |
| Elongation at break(pure_240H) | Purine nucleosides<br>Carboxylic acids and derivatives<br>Carboxylic acids and derivatives<br>Carboxylic acids and derivatives<br>Carboxylic acids and derivatives |
| M50(pure_500H) | Piperidines<br>Pyrimidine nucleosides<br>Pyridines and derivatives<br>Carboximidic acids and derivatives<br>Carboxylic acids and derivatives<br>Organooxygen compounds |

| measured items | Classes of each metabolite selected in sequential selection model |
|---|---|
| M100(pure_500H) | Carboxylic acids and derivatives<br>Glycerophospholipids<br>Carboxylic acids and derivatives<br>Carboximidic acids and derivatives<br>Carboxylic acids and derivatives<br>Carboxylic acids and derivatives |
| M200(pure_500H) | Carboxylic acids and derivatives<br>Carboxylic acids and derivatives<br>Purine nucleosides<br>Purine nucleosides<br>Carboxylic acids and derivatives |
| Tensile stress(pure_500H) | Phenols<br>Fatty Acyls<br>Carboximidic acids and derivatives<br>Ribonucleoside 3'-phosphates<br>Carboxylic acids and derivatives<br>Organonitrogen compounds |
| Elongation at break(pure_500H) | Carboxylic acids and derivatives<br>Sulfinic acids and derivatives<br>Prenol lipids<br>Steroids and steroid derivatives<br>Azoles |
| M50(carbon_72H) | Pyrimidine nucleosides<br>Quinolines and derivatives<br>Benzene and substituted derivatives<br>Organooxygen compounds<br>Carboxylic acids and derivatives<br>Fatty Acyls |

| measured items | Classes of each metabolite selected in sequential selection model |
|---|---|
| M100(carbon_72H) | Glycerophospholipids<br>Carboxylic acids and derivatives<br>Fatty Acyls<br>Carboxylic acids and derivatives<br>Carboxylic acids and derivatives |
| M200(carbon_72H) | Carboxylic acids and derivatives<br>Fatty Acyls<br>Carboxylic acids and derivatives<br>Carboxylic acids and derivatives<br>Hydroxy acids and derivatives |
| M300(carbon_72H) | Glycerophospholipids<br>Fatty Acyls<br>Glycerophospholipids<br>Carboxylic acids and derivatives<br>Carboxylic acids and derivatives |
| Tensile stress(carbon_72H) | Organonitrogen compounds<br>Organooxygen compounds<br>Fatty Acyls<br>Fatty Acyls<br>Benzene and substituted derivatives |
| Elongation at break(carbon_72H) | Carboxylic acids and derivatives<br>Carboxylic acids and derivatives<br>Carboxylic acids and derivatives<br>Carboxylic acids and derivatives<br>Carboxylic acids and derivatives |
| M50(carbon_240H) | Organonitrogen compounds<br>Azoles<br>Fatty Acyls<br>Organic carbonic acids and derivatives<br>Sphingolipids |

| measured items | Classes of each metabolite selected in sequential selection model |
|---|---|
| M100(carbon_240H) | Fatty Acyls<br>Organonitrogen compounds<br>Fatty Acyls<br>Organic carbonic acids and derivatives<br>Pyridines and derivatives |
| M200(carbon_240H) | Pyrimidine nucleosides<br>Carboxylic acids and derivatives<br>Benzene and substituted derivatives<br>Organonitrogen compounds<br>Organooxygen compounds |
| M300(carbon_240H) | Carboxylic acids and derivatives<br>Fatty Acyls<br>Carboximidic acids and derivatives<br>Organonitrogen compounds<br>Carboxylic acids and derivatives<br>Carboxylic acids and derivatives |
| Tensile stress(carbon_240H) | Organonitrogen compounds<br>Carboxylic acids and derivatives<br>Glycerophospholipids<br>Carboxylic acids and derivatives<br>Steroids and steroid derivatives<br>Prenol lipids |
| Elongation at break(carbon_240H) | Organonitrogen compounds<br>Pteridines and derivatives<br>Carboxylic acids and derivatives<br>Carboxylic acids and derivatives<br>Organonitrogen compounds<br>Fatty Acyls |

| **measured items** | **Classes of each metabolite selected in sequential selection model** |
|---|---|
| M50(carbon_500H) | Fatty Acyls<br>Carboximidic acids and derivatives<br>Fatty Acyls<br>Steroids and steroid derivatives<br>Organonitrogen compounds<br>Organonitrogen compounds |
| M100(carbon_500H) | Organonitrogen compounds<br>Fatty Acyls<br>Carboximidic acids and derivatives<br>Indoles and derivatives<br>Steroids and steroid derivatives<br>Organonitrogen compounds |
| M200(carbon_500H) | Phenols<br>Organonitrogen compounds<br>Carboxylic acids and derivatives<br>Fatty Acyls<br>Azoles<br>Fatty Acyls |
| Tensile stress(carbon_500H) | Sulfinic acids and derivatives<br>Carboxylic acids and derivatives<br>Piperidines<br>Organonitrogen compounds<br>Azoles |
| Elongation at break(carbon_500H) | Fatty Acyls<br>Pyrroles<br>Carboxylic acids and derivatives<br>Organooxygen compounds<br>Hydroxy acids and derivatives |

**Supplementary Table 4:** Items measured in characterization of NR and the measurement methods used. The measurement method basically follows the JIS (Japanese Industrial Standards) below. Any changes from the JIS are noted.

| JIS | Notation of measurement iems in the text | Explanation |
|---|---|---|
| JIS K6300-1 | M1+3(raw) | Mooney viscosity after 1 minute of pre-heating and 3 minutes of measurement |
| JIS K6300-1 | Vm(raw) | Minimum Mooney viscosity |
| JIS K6300-1 | M1+3(pure) | Mooney viscosity after 1 minute of pre-heating and 3 minutes of measurement |
| JIS K6300-1 | Vm(pure) | Minimum mooney viscosity |
| JIS K6300-1 | st5(pure) | Time elapsed when Mooney viscosity increased by 5 units |
| JIS K6300-1 | st10(pure) | Time elapsed when Mooney viscosity increased by 10 units |
| JIS K6300-1 | st10-st5(pure) | Difference between st10 and st5 |
| JIS K6300-2 | MH(pure) | Maximum torque |
| JIS K6300-2 | ML(pure) | Minimum torque |
| JIS K6300-2 | s0.4(pure) | Time it takes for torque to increase by 0.4 from the minimum value |
| JIS K6300-2 | T10(pure) | Time when vulcanization has progressed 10% |
| JIS K6300-2 | T50(pure) | Time when vulcanization has progressed 50% |
| JIS K6300-2 | T90(pure) | Time when vulcanization has progressed 90% |
| JIS K6300-2 | T90-T10(pure) | Difference between T90 and T10 |

| JIS | Notation of measurement iems in the text | Explanation |
|---|---|---|
| JIS K6300-2 | tMH(pure) | Time when maximum torque was measured |
| JIS K6300-2 | tML(pure) | Time when minimum torque was measured |
| JIS K6300-1 | M1+3(carbon) | Mooney viscosity after 1 minute of pre-heating and 3 minutes of measurement |
| JIS K6300-1 | Vm (carbon) | Minimum Mooney viscosity |
| JIS K6300-1 | st5p(carbon) | Time elapsed when Mooney viscosity increased by 5 units |
| JIS K6300-1 | st10(carbon) | Time elapsed when Mooney viscosity increased by 10 units |
| JIS K6300-1 | st10-st5(carbon) | Difference between st10 and st5 |
| JIS K6300-2 | MH(carbon) | Maximum torque |
| JIS K6300-2 | ML(carbon) | Minimum torque |
| JIS K6300-2 | s0.4(carbon) | Time it takes for torque to increase by 0.4 from the minimum value |
| JIS K6300-2 | T10(carbon) | Time when vulcanization has progressed 10% |
| JIS K6300-2 | T50(carbon) | Time when vulcanization has progressed 50% |
| JIS K6300-2 | T90(carbon) | Time when vulcanization has progressed 90% |
| JIS K6300-2 | T90-T10(carbon) | Difference between T90 and T10 |
| JIS K6300-2 | tMH(carbon) | Time when maximum torque was measured |
| JIS K6300-2 | tML(carbon) | Time when minimum torque was measured |

| JIS | Notation of measurement iems in the text | Explanation |
|---|---|---|
| JIS K6251 | M25(pure_0H) | Stress when gauge length is stretched by 25% |
| JIS K6251 | M50(pure_0H) | Stress when gauge length is stretched by 50% |
| JIS K6251 | M100(pure_0H) | Stress when gauge length is stretched by 100% |
| JIS K6251 | M200(pure_0H) | Stress when gauge length is stretched by 200% |
| JIS K6251 | M300(pure_0H) | Stress when gauge length is stretched by 300% |
| JIS K6251 | Tensile stress(pure_0H) | The stress at which the sample breaks |
| JIS K6251 | Elongation at break(pure_0H) | Elongation of gauge length at which the sample breaks |
| JIS K6251 | M25(carbon_0H) | Stress when gauge length is stretched by 25% |
| JIS K6251 | M50(carbon_0H) | Stress when gauge length is stretched by 50% |
| JIS K6251 | M100(carbon_0H) | Stress when gauge length is stretched by 100% |
| JIS K6251 | M200(carbon_0H) | Stress when gauge length is stretched by 200% |
| JIS K6251 | M300(carbon_0H) | Stress when gauge length is stretched by 300% |
| JIS K6251 | Tensile stress(carbon_0H) | The stress at which the sample breaks |
| JIS K6251 | Elongation at break(carbon_0H) | Elongation of gauge length at which the sample breaks |
| JIS K6257, K6251 | M50(pure_72H) | Stress when gauge length is stretched by 50% after 72 hours of heat aging |

| JIS | Notation of measurement iems in the text | Explanation |
|---|---|---|
| JIS K6257, K6251 | M100(pure_72H) | Stress when gauge length is stretched by 100% after 72 hours of heat aging |
| JIS K6257, K6251 | M200(pure_72H) | Stress when gauge length is stretched by 200% after 72 hours of heat aging |
| JIS K6257, K6251 | M300(pure_72H) | Stress when gauge length is stretched by 300% after 72 hours of heat aging |
| JIS K6257, K6251 | Tensile stress(pure_72H) | The stress at which the sample breaks after 72 hours of heat aging |
| JIS K6257, K6251 | Elongation at break(pure_72H) | Elongation of gauge length at which the sample breaks after 72 hours of heat aging |
| JIS K6257, K6251 | M50(pure_240H) | Stress when gauge length is stretched by 50% after 240 hours of heat aging |
| JIS K6257, K6251 | M100(pure_240H) | Stress when gauge length is stretched by 100% after 240 hours of heat aging |
| JIS K6257, K6251 | M200(pure_240H) | Stress when gauge length is stretched by 200% after 240 hours of heat aging |
| JIS K6257, K6251 | M300(pure_240H) | Stress when gauge length is stretched by 300% after 240 hours of heat aging |
| JIS K6257, K6251 | Tensile stress(pure_240H) | The stress at which the sample breaks after 240 hours of heat aging |
| JIS K6257, K6251 | Elongation at break(pure_240H) | Elongation of gauge length at which the sample breaks after 240 hours of heat aging |

| JIS | Notation of measurement iems in the text | Explanation |
|---|---|---|
| JIS K6257, K6251 | M50(pure_500H) | Stress when gauge length is stretched by 50% after 500 hours of heat aging |
| JIS K6257, K6251 | M100(pure_500H) | Stress when gauge length is stretched by 100% after 500 hours of heat aging |
| JIS K6257, K6251 | M200(pure_500H) | Stress when gauge length is stretched by 200% after 500 hours of heat aging |
| JIS K6257, K6251 | Tensile stress(pure_500H) | The stress at which the sample breaks after 500 hours of heat aging |
| JIS K6257, K6251 | Elongation at break(pure_500H) | Elongation of gauge length at which the sample breaks after 500 hours of heat aging |
| JIS K6257, K6251 | M50(carbon_72H) | Stress when gauge length is stretched by 50% after 72 hours of heat aging |
| JIS K6257, K6251 | M100(carbon_72H) | Stress when gauge length is stretched by 100% after 72 hours of heat aging |
| JIS K6257, K6251 | M200(carbon_72H) | Stress when gauge length is stretched by 200% after 72 hours of heat aging |
| JIS K6257, K6251 | M300(carbon_72H) | Stress when gauge length is stretched by 300% after 72 hours of heat aging |
| JIS K6257, K6251 | Tensile stress(carbon_72H) | The stress at which the sample breaks after 72 hours of heat aging |
| JIS K6257, K6251 | Elongation at break(carbon_72H) | Elongation of gauge length at which the sample breaks after 72 hours of heat aging |

| JIS | Notation of measurement iems in the text | Explanation |
|---|---|---|
| JIS K6257, K6251 | M50(carbon_240H) | Stress when gauge length is stretched by 50% after 240 hours of heat aging |
| JIS K6257, K6251 | M100(carbon_240H) | Stress when gauge length is stretched by 100% after 240 hours of heat aging |
| JIS K6257, K6251 | M200(carbon_240H) | Stress when gauge length is stretched by 200% after 240 hours of heat aging |
| JIS K6257, K6251 | M300(carbon_240H) | Stress when gauge length is stretched by 300% after 240 hours of heat aging |
| JIS K6257, K6251 | Tensile stress(carbon_240H) | The stress at which the sample breaks after 240 hours of heat aging |
| JIS K6257, K6251 | Elongation at break(carbon_240H) | Elongation of gauge length at which the sample breaks after 240 hours of heat aging |
| JIS K6257, K6251 | M50(carbon_500H) | Stress when gauge length is stretched by 50% after 500 hours of heat aging |
| JIS K6257, K6251 | M100(carbon_500H) | Stress when gauge length is stretched by 100% after 500 hours of heat aging |
| JIS K6257, K6251 | Tensile stress(carbon_500H) | The stress at which the sample breaks after 500 hours of heat aging |
| JIS K6257, K6251 | Elongation at break(carbon_500H) | Elongation of gauge length at which the sample breaks after 500 hours of heat aging |

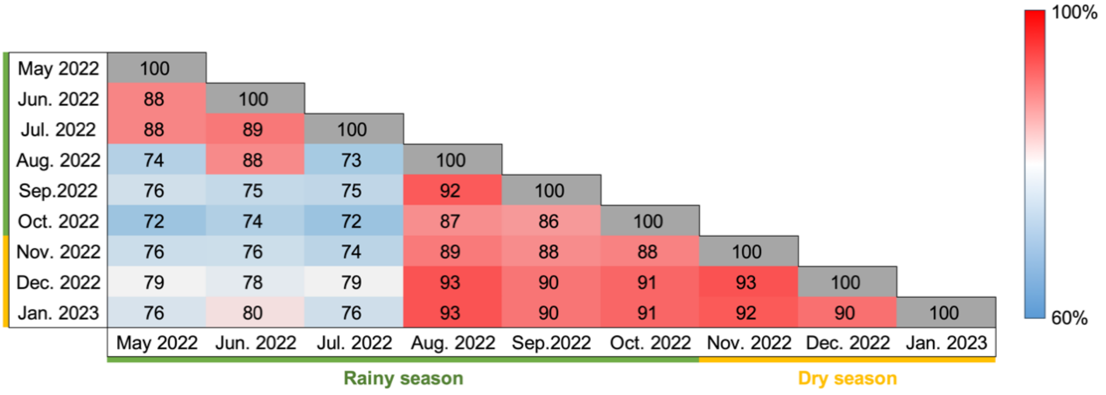


**Supplementary Fig. 1:** Percentage of common peaks among the monthly results of metabolite analysis.

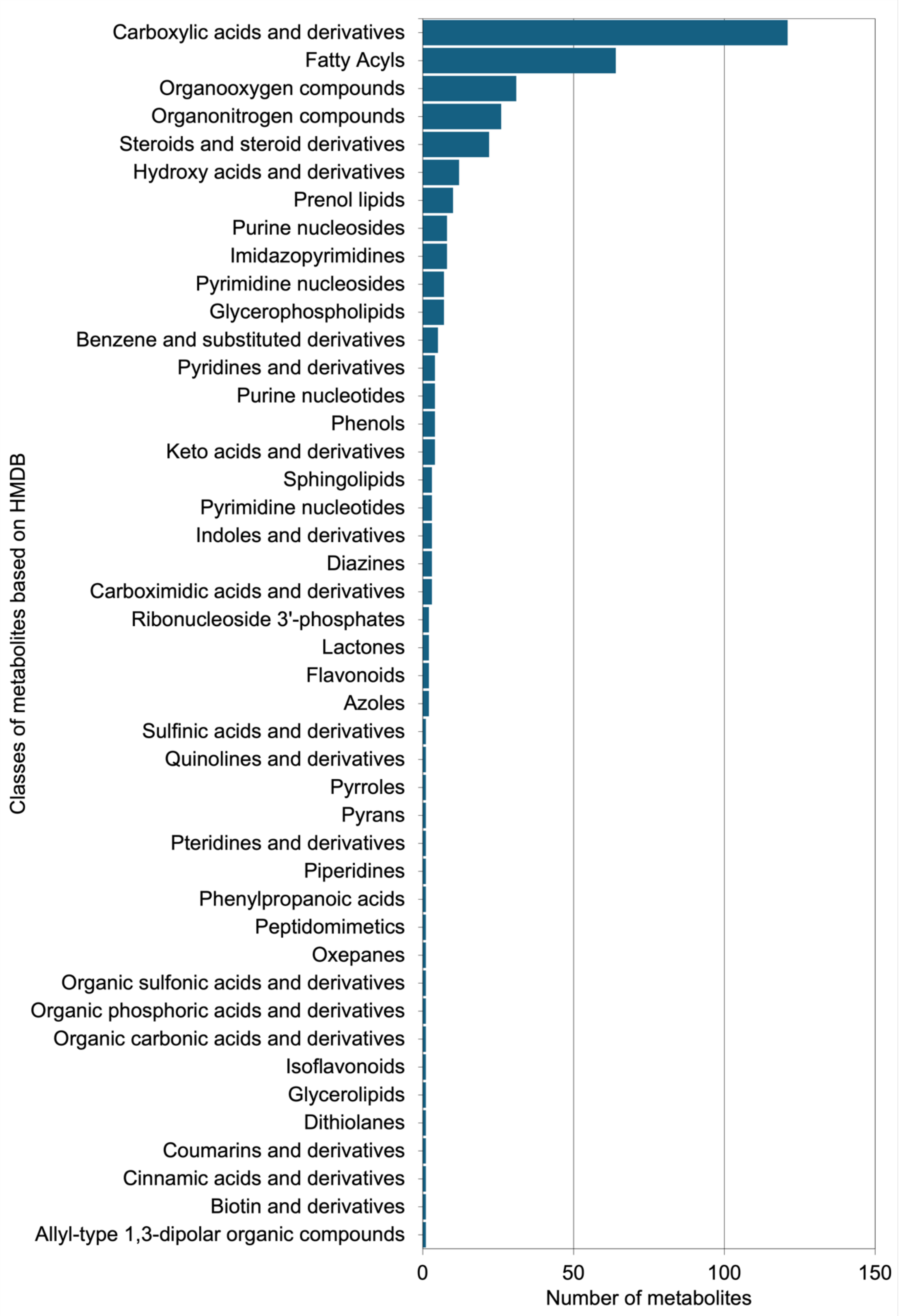


**Supplementary Fig. 2:** Classes of detected metabolites based on chemical taxonomy information from Human Metabolome Database.

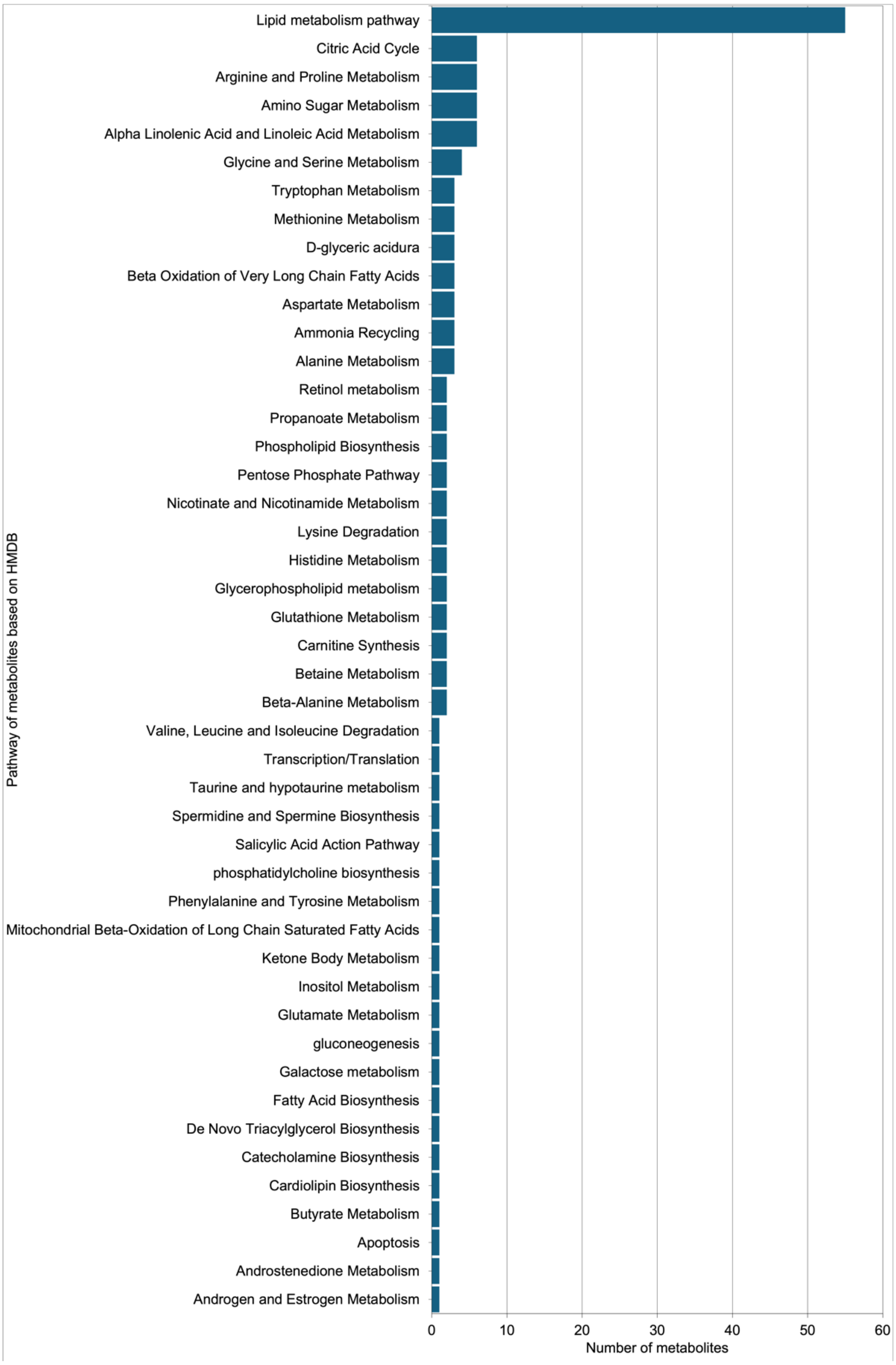


**Supplementary Fig. 3:** Metabolic pathways the detected metabolites belong to, according to metabolic pathway information from the Human Metabolome Database.

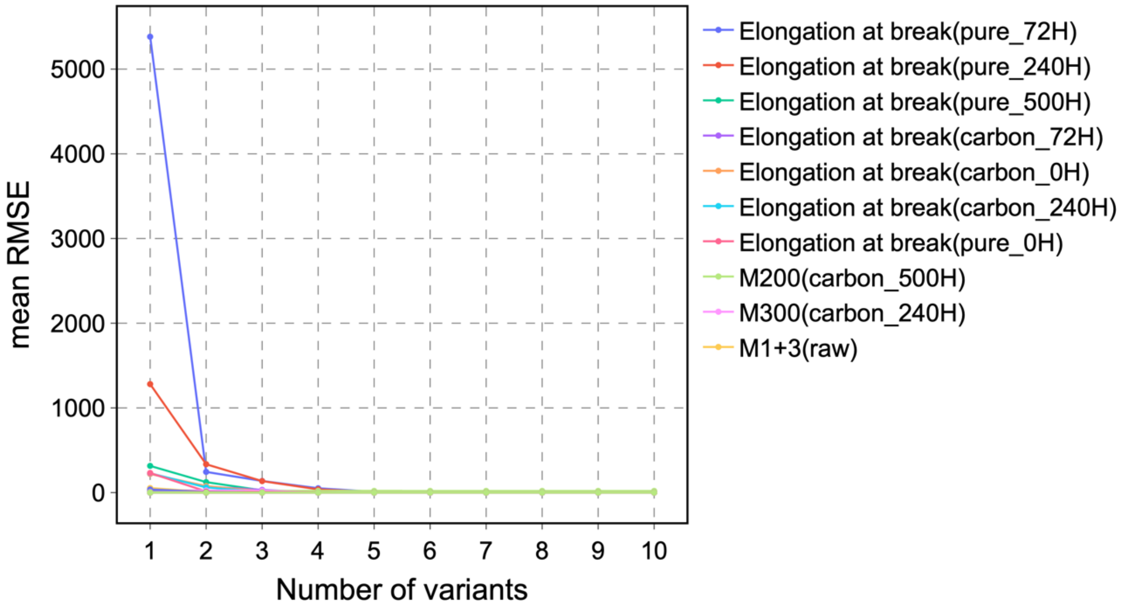


**Supplementary Fig. 4:** Transition of error values during the variable selection process in the sequential selection model, the legend shows only the top 10 average error values when there are 1 to 10 variables.
